# Drug Delivery to the Brain: Recent Advances and Unmet Challenges

**DOI:** 10.3390/pharmaceutics15122658

**Published:** 2023-11-23

**Authors:** Sukanya Bhunia, Nagesh Kolishetti, Arti Vashist, Adriana Yndart Arias, Deborah Brooks, Madhavan Nair

**Affiliations:** 1 Department of Immunology and Nano-Medicine, Herbert Wertheim, College of Medicine, Florida International University, Miami, FL 33199, USA; 2 Institute of Neuroimmune Pharmacology, Herbert Wertheim College of Medicine, Florida International University, Miami, FL 33199, USA

**Keywords:** blood–brain barrier (BBB), focus ultrasound, nanocarrier, drug delivery to the brain, receptor-mediated transcytosis, brain tumor, neurodegenerative disease

## Abstract

Brain cancers and neurodegenerative diseases are on the rise, treatments for central nervous system (CNS) diseases remain limited. Despite the significant advancement in drug development technology with emerging biopharmaceuticals like gene therapy or recombinant protein, the clinical translational rate of such biopharmaceuticals to treat CNS disease is extremely poor. The blood–brain barrier (BBB), which separates the brain from blood and protects the CNS microenvironment to maintain essential neuronal functions, poses the greatest challenge for CNS drug delivery. Many strategies have been developed over the years which include local disruption of BBB via physical and chemical methods, and drug transport across BBB via transcytosis by targeting some endogenous proteins expressed on brain-capillary. Drug delivery to brain is an ever-evolving topic, although there were multiple review articles in literature, an update is warranted due to continued growth and new innovations of research on this topic. Thus, this review is an attempt to highlight the recent strategies employed to overcome challenges of CNS drug delivery while emphasizing the necessity of investing more efforts in CNS drug delivery technologies parallel to drug development.

## 1. Introduction

Despite the recent advances in genomics and neurobiology, central nervous system (CNS) diseases from brain tumors to neurological diseases continue to remain a global concern due to its complex protective structure [1]. As per the estimation of WHO in 2016, one-third of the global population is impacted by neurological or psychiatric conditions at some point in their lifetime with Alzheimer’s disease (AD) alone is estimated to cost US$2 trillion by 2030 [2]. In addition, brain tumor remains to be the most diagnosed solid tumor in children and adolescents and the leading cause of cancer death among young adults [3]. In spite of the advancement of drug discovery technologies, drug development for CNS diseases remains a formidable task with a probability of only a few percentage (~8.2%) of drugs developed to be translated for clinical use [4]. The heterogeneity of CNS diseases and the lack of proper preclinical models to accurately mimic human pathology play crucial roles, these challenges of drug delivery to the brain are the major factors behind the poor clinical translation rate of CNS drugs.

Introducing drugs into the brain poses a unique set of challenges compared to other body tissues due to protective mechanisms that safeguard brain tissue externally and internally. Externally, the brain is protected by the skull and three inner layers of membranes known as meninges which regulate intracranial tissue pressure by constraining the volume [5] while it is cushioned by cerebrospinal fluid (CSF) that flows within the meninges and acts as a shock absorber to protect the brain from injury. Internally, the brain benefits from the presence of the blood–brain barrier (BBB). BBB prevents random entry of solutes, including bloodborne pathogens and neurotoxins to maintain the highly regulated CNS microenvironment essential for neuronal functions while permitting the exchange of nutrients and metabolic waste to maintain brain homeostasis. The intracranial tissue pressure in addition to the hurdle of invasive drilling of the skull largely limits the scope of local drug delivery to the brain while the systemic drug delivery to the brain is greatly hampered by the blood–brain barrier which rejects ~98% of substances. Biopharmaceuticals such as recombinant proteins or monoclonal antibodies (mAb) which have emerged as a promising part of drug development in the past two decades have failed in treating CNS disease due to their poor access to the brain across the BBB. For instance, Bevacizumab (Avastin) [6] and Natalizumab (Tysabri) [7], which are FDA approved monoclonal antibody-based therapeutics for treating brain cancer and multiple sclerosis, respectively, do not cross the BBB [8]. Thus, there is an urgent need for efficient technology that can effectively deliver pharmaceuticals to brain with minimum adverse effect. To this end, several strategies have been developed and evaluated in pre-clinical and clinical settings over the past decades, but none of these have yet turned out to be groundbreaking.

Herein, we have provided a overview on the structural aspects of the blood–brain barrier limiting the systemic drug delivery to the brain and discussed the drug delivery strategies to overcome it with a focus on the physical and cellular stimulation of BBB for enhanced permeation of pharmaceutics across BBB. In addition, we have discussed the localized drug delivery strategies for getting drugs into the brain and finally, have shed light on the importance of research efforts not only to drug development but also to delivery strategies for brain.

## 2. Structure and Function of Blood–Brain barrier 

**Figure 1:**
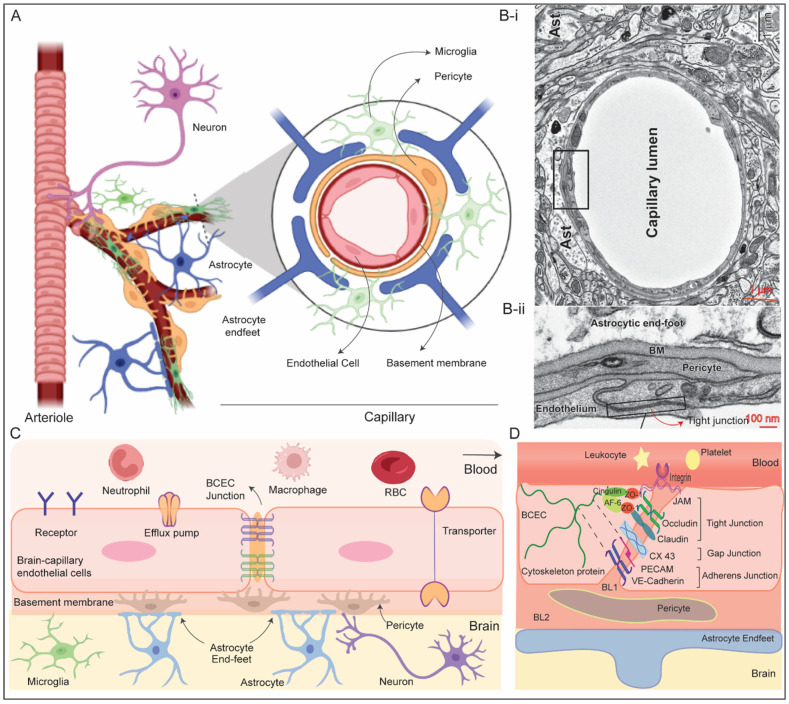
(**A**) Schematic representation of the neurovascular unit (NVU) comprised of neurons, vascular cells (endothelial cells-EC, smooth muscle cells-SMC at arterioles, and pericytes-PC at capillary), glial cells (astrocytes-Ast and microglia). EC are covered by PC and astrocyte end-feet which are embedded in the basement membrane (BM). Neurons communicate with adjacent mural cells (PC and SMC) to regulate blood flow in the brain microvasculature. Microglia lies around the open area of brain capillary that is not covered by astrocyte end-feet. (**B-i**) Transmission electron micrograph (TEM) of brain tissue depicting NVU (**B-ii**) component EC, PC, Ast, and tight junction; Adopted with permission from Ref. [1] (**C**) schematic of blood–brain barrier comprising interconnected brain capillary endothelial cells via junction proteins (**D**) along with the other cellular components of NVU.

BBB is an endothelial membrane barrier within brain microvascular formed by tight junction of brain capillary endothelial cells (BCEC) sheathed by mural cells and astrocytes end-feet that separate CNS from systemic blood circulation (Figure 1A,B) [9,10]. The BBB protects the brain by preventing random entry of substances like neurotoxins and bloodborne pathogens while maintaining the brain homeostasis by selective passage of nutrients. BBB is composed of a continuous layer of BCEC tightly connected by junction proteins sheathed by mural cells (pericyte at the microcapillary and vascular smooth muscle cells in arteries and arterioles), basement membrane, glial cells (astrocyte, microglia and oligodendrocyte) and neurons which are together known as neurovascular unit (NVU) [1,11]. Cellular components of NVU functionally interact to maintain integrity of microvasculature including BBB and regulate cerebral blood flow. The CNS microvascular differs from the peripheral microvascular, the former can be extremely thin (thickness ~200 nm) and the intercellular junction of BCEC is ~50–100 times tighter compared to the peripheral. In addition, there is no fenestration in BCEC with limited number of pinocytotic vesicles unlike peripheral blood endothelial cells. This requires energy dependent active transport pathway for nutrient transport across BBB which further supports the presence of ~5–6 times more mitochondria in BCEC. Furthermore, presence of proteolytic enzymes capable of rupturing neuroactive bloodborne solutes and drugs in BCEC offers an additional enzymatic barrier [11].

Pericytes are embedded in vascular basement membrane and cover approximately 20% of abluminal (outer) surface of the BBB. These cells possess contractile proteins, allowing them to regulate blood flow in brain capillaries through contraction and relaxation. There are two basal lamina basement membranes (BMs), the inner vascular BM is formed by the extracellular matrix (ECM) secreted by BCEC and pericytes and the outer parenchymal BM is formed by secreted ECM from astrocytic process. The BMs anchor for signaling process and acts as an additional barrier. Astrocytes, a major glial cell type, play essential role in maintaining structure and function of the BBB [12]. The end-feet of astrocytes create an intricate network that surrounds BCECs, strengthening the tight junctions, almost completely ensheathe the brain endothelial capillary and maintain structural integrity of the BBB. It also connects BCEC with neurons and mediate inter-cellular communication to regulate vascular contraction/dilution and blood flow in response to neuronal response [12]. In addition, astrocytes play crucial role in maintaining brain homeostasis, clearing synapses, injury protection and considered as the primary workhorse of the CNS for such versatile roles [13]. Microglia mediates immune regulation in brain and plays crucial role in maintaining CNS homeostasis. In addition, recent studies support that activated microglia can increase the expression of tight junction proteins [14].

Three types of junctions, namely, tight junctions (TJ), adherens junctions (AJ), and gap junctions play role behind the extremely tight connection among adjacent BCEC (Figure 1C,D). Tight junctions contains both transmembrane proteins such as Junction adhesion molecules (JAMs), claudins, occludins, etc., and cytoplasmic proteins like zonula occludins (ZO), afadin (e.g., AF-6), cingulin, etc. [15] Claudins (~27 kDa) is the most crucial transmembrane tight junction proteins within the BBB. Claudins extracellular segments create TJ that tightly seal the space between neighboring BCECs, while their intracellular domains connect with actin filaments (Figure 1D). Occludins is another transmembrane protein exclusively localized at the tight junctions and perform similar function like claudins. JAMs are expressed in tight junction of BCEC regulate migration of leukocyte and platelet via integrin receptor based adhesive interaction. The cytoplasmic domain of TJs interacts with cytoskeletal and the basal adherens junction proteins which are also essential to maintain barrier property of the BBB.

The Adherens junction, which is formed by homodimeric transmembrane cadherin protein at the basal side, is important for proper assembly of tight junction proteins and structural integrity of the BBB [16]. The extracellular domain of vascular endothelial (VE)-cadherin of one BCEC span across the paracellular cleft to dimerize with another extracellular domain of VE-cadherin from neighboring BCEC to provide the structural support while the cytoplasmic domains are connected to actin filament via catenin proteins. Stabilization of catenin induces expression of claudin-3 which supports assembly of the tight junction. Platelet EC adhesion molecule 1 (PECAM-1), nectin, CD99 are other transmembrane protein components of adherens junction, and their roles are still under investigation. Gap junction, which structurally mimic an intercellular channel formed by hexamer of integral proteins connexins and pannexins (e.g., Cx37, Cx40, Cx43) connecting to adjacent endothelial cells, is located between the TJs and AJs [17]. It permits the exchange of ions, small metabolites, and signals between adjacent BCEC and plays important role in maintaining homeostasis of the BBB. Furthermore, it governs the permeability by engaging with cytoplasmic proteins like ZO-1 through afadin-6 protein. In combination, the presence of these particular endothelial junctions, especially TJ, markedly hinders the transit of random substances across the BBB. The encapsulation of brain endothelial cell capillaries by the astrocytes and pericytes further contributes to the tightness of the BBB which can be estimated by a parameter known as transendothelial electrical resistance (TEER) as 1500–2000 Ω cm^2^ [18].

The BBB undergoes structural and functional alteration in CNS diseases which often compromises its structural integrity. For instance, in the context of brain tumors, BBB is referred as blood–tumor barrier (BTB) and it exhibits distinct features, including the loss of junctional proteins in endothelial cells, aberrant distribution of pericyte, loss of astrocytic endfeet and neuronal connections, and an increased infiltration of circulating immune cells into glioma tissue [19,20]. Furthermore, with the progression of tumor, vascularization greatly hampered the structural integrity of BBB. With an average-sized tumor, approximately 10% of the BBB may display open junctions, while around 30% may develop fenestrations that allow the passage of molecules up to 330 kDa in size [21,22]. It is important to note that, despite the disruption in the core of the tumor, the BBB may still maintain its barrier properties intact in other areas of brain. The BTB shares common characteristics with the BBB, including the expression of efflux transporters in endothelial cells and tumor cells. Additionally, the BTB often exhibit higher expression of certain receptors that promote tumor growth, such as GLUT1 and BCRP [23]. Similar pathological breakdown of barrier property is also observed in neurological diseases [10]. For leukocytes, altered expression of ion-channel receptors, and transporters are also observed that compromises protective function of the BBB.

## 3. Approaches for Drug Delivery through the Blood–Brain Barrier

**Figure 2:**
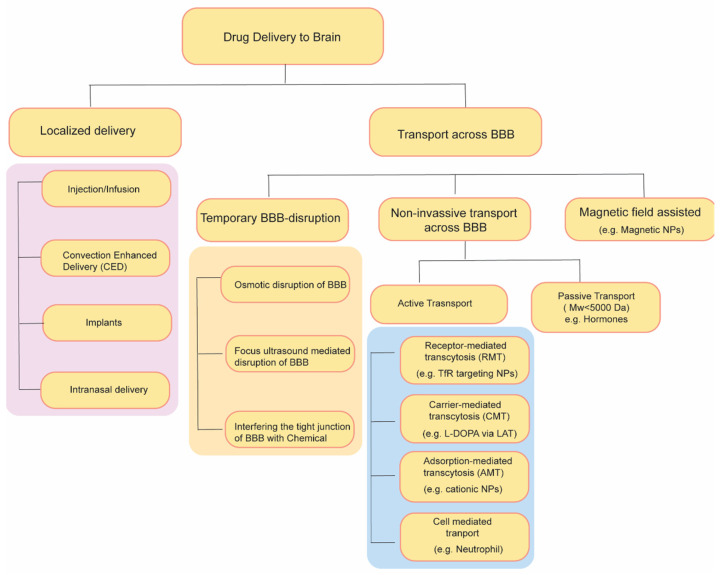
Summary of drug delivery strategies to brain.

Over the past few decades, diverse strategies have emerged to enhance the transportation of drugs through the BBB (Figure 2). These strategies include temporary disruption of BBB via physical or chemical means as well as targeting some endogenous transporter systems over-expressed on BBB.

### 3.1. Temporary Disruption of BBB

#### 3.1.1. Osmotic Blood–Brain Barrier Disruption

**Figure 3:**
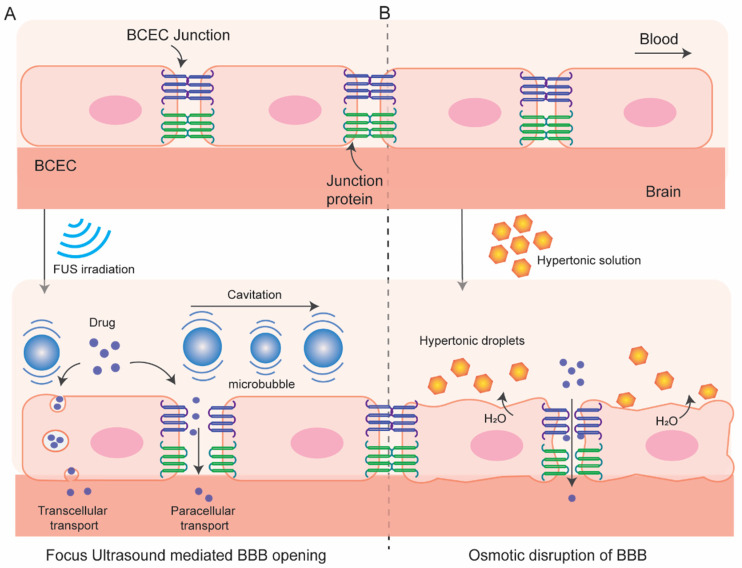
Schematic of focus ultrasound-mediated (**A**) and osmotic (**B**) disruption of BBB.

In this process (Figure 3), BBB permeation is achieved using a hyperosmotic agent which causes dehydration and shrinkage in BCEC resulting tight-junction dysfunction and transient disruption of BBB. This process of osmotic BBB disruption was first hypothesized by Rapoport et al. in 1972 [24] following an improved BBB permeation of a dye Evan’s blue when co-administered with hypertonic arabinose and later supported by Brightman et al. who visualize the opening of tight junction with electron microscopy after intra-carotid infusion of mannitol [25]. A variety of substances have been used as osmotic disruptors of the BBB including urea, lactamide, saline but mannitol has been most used for this purpose. Since 1980, intracarotid artery hyperosmotic mannitol (ICAHM) infusions has been used for drug delivery to brain in several pre-clinical and clinical studies [26] many of which have produced encouraging results of enhanced survivability with clinical safety. For instance, a clinical study conducted in 17 patients with primary CNS lymphoma receiving cyclophosphamide and mannitol followed by radiotherapy significantly enhances the mean survivability (from 17.8 months to 44.5 months) compared with the control group receiving radiotherapy alone [27]. Combination of carboplatin and etoposide delivered in this method exhibits an effective delivery in brain and dramatic responses in inhibiting CNS tumor in patients although unexpected high-frequency hearing loss limits the application of the combined chemotherapy [28]. Some studies in animal models have demonstrated variable and inconsistent results in BBB permeability like nonselective opening of BBB, CNS toxicity and neuroinflammatory response become the major limitation of this approach [29,30,31]. The success of this strategy depends on multiple factors, including injection speed, optimum mannitol dose, cerebral hemodynamics, and vascular anatomy. Strategies to overcome the limitation are currently under investigation, e.g., use of real time MRI guidance for optimum and targeted delivery of therapeutics [32,33]. Overall, mannitol mediated osmotic disruption of BBB for drug delivery to brain is safe and hold promise while further investigation is needed to improve its reproducibility and clinical effectiveness.

#### 3.1.2. BBB Disruption with Focused Ultrasound

In this method (Figure 3) local BBB permeation can be achieved by using focused ultrasound (FUS) in combination with intravenous microbubbles and can be monitored by using MR-imaging system. This method has several advantages over other methods. It is reproducible, non-invasive, and targeted opening of the BBB can be achieved. In addition, the BBB opening is transient which can be restored within 6 to 24 h allowing accumulation of therapeutics in the region of interest for a desired time window.

The FUS technology was first introduced in 1950s initially to treat psychiatric disorders and brain tumors although those early attempts were invasive involving craniectomy to introduce sonication into brain which has been evolved to non-invasive over time by decades of research [34,35,36,37]. Although the minimal invasiveness to reduce surgical trauma and recovery time are the driving force, the skull bone which varies in thickness and density among individuals greatly attenuates and distorts ultrasound. In addition, hair, which introduces air, significantly (up to 80%) distorts the delivery of ultrasound. The implementation of phased array transducers along with real-time MRI-thermal monitoring has been a breakthrough in this century to made non-invasive transcranial FUS feasible [37].

The cellular and molecular mechanism of FUS-mediated enhanced BBB permeation is poorly understood. The sheer stress from the stable acoustic cavitation of the microbubbles induces structural and functional modulation in the BBB like higher caveolae formation, sonoporation, as well as opening of some tight junctions which enhance intracellular and paracellular transport [38,39,40]. Although stable cavitation contributes to loosening of tight junction, inertial cavitation may contribute to hemorrhage and ischemia. Nonetheless, microbubble cavitation can be controlled by tuning ultrasound pressure amplitude and low-frequency FUS-mediated BBB opening rule out the thermal effect on the BBB. Notably, FUS activates PI3kinase/Akt pathway in neuronal cells which may play role in modulation of tight junction proteins ZO-1 and occludin [41]. Cerebral vessels are resilient to such mechanical stress caused by stable microbubbles cavitation and quickly recover their integrity after the FUS.

As indicated before, FUS can induce local and targeted opening of the BBB with a desired time window. The extent of BBB opening can be controlled by tuning ultrasound pressure amplitude, transducer frequency, microbubble size and dosage, exposure duration and burst parameters [42,43,44]. For instance, a study by Chen et al. has demonstrated that FUS can enable trans-BBB delivery of dextran molecule up to 2000 kDa (hydrodynamic diameter 2.3 to 54.4 nm) at a 0.84 MPa acoustic pressure [45]. However, small opening (70 kDa) can be achieved by stable cavitation whereas larger BBB opening (>500 kDa) is associated with inertial cavitation. Thus, FUS has been demonstrated to markedly enhance the trans-BBB delivery of therapeutic antibodies (~150 kDa, e.g., Herceptin) [7,44,46,47,48], chemotherapeutics [49], and cells [50,51,52] and shows clinical promise in treating brain tumor and other CNS diseases [37,53]. Furthermore, studies indicate that FUS can be utilized to target therapeutics in different regions of the brain such as the hippocampus [54], striatum [55], cortical targets [46], and brainstem [49]. The safety of FUS-mediated BBB opening is promising. A mild and short term (<2 weeks) immune response is reported after repeated administration [56,57,58,59]. Importantly, behavioral, morphological, and neuroimaging characteristics are retained even after long-term repeated administration of FUS in animal models (biweekly over 6 months in rats or 4 months in non-human primates) [60,61].

#### 3.1.3. Radiation-Mediated BBB Disruption

Few studies have reported that radiation therapy, an important modality in treating brain tumor, may play a role in disrupting the BBB and enhance drug entry to brain [62]. However, the role of radiation in increasing drug accumulation in brain and its underlying mechanisms are still uncertain. In addition, radiation induced BBB disruption is not temporary and the recovery time is much higher (in years) which often lead to radiation induced toxicity including headache, neurologic deficits or nausea [63].

#### 3.1.4. Interfering the Tight Junction of BBB with Chemicals

Disengaging the tight junctions of BBB is another strategy to improve permeability across BBB. Bradykinin (BK), a peptide containing 9 aminoacids upon administration causes dilation of arterioles and enhances paracellular transport by down-regulating expression of the tight junction proteins (occluding, ZO-1, and claudin-5) and improves transcellular transport by upregulating caveolin mediated pinocytotic vesicles [64]. The BBB opening potential of bradykinin, and its synthetic analogs, have been explored [65,66,67] especially in brain tumors due to the high expression of BK receptor at BTB [68]. However, it did not go through Phase-II mainly because the extremely transient opening of BBB and the adverse side effects due to the wide distribution of BK receptors at numerous additional sites beyond the BBB [69]. BBB disruption via targeting claudin-5, a major component of BBB tight junctions, via siRNA mediated knockdown or using anti-claudin5-antibody also demonstrated to enhance BBB permeation transiently and reversibly [70,71]. It also suffers similar limitations of transient effects and adverse side effects due to wider distribution of receptor expression. To this end, targeting Angulin-1, another functional constituent of BBB tricellular tight junctions which is majorly expressed in BBB and selectively blocks entry of macromolecules into the brain, can address the adverse effects [72]. Angubindin-1, a ligand of angulin-1, is demonstrated to enhance the entry of macromolecules across BBB by removing angulin-1 and disrupting the tricellular tight junctions [73].

### 3.2. Drug Transport without Disrupting BBB: Active and Passive Transport Pathways

**Figure 4:**
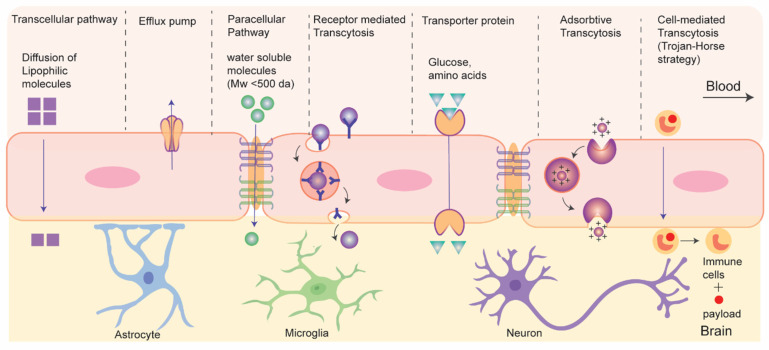
Schematics for drug transport pathways across BBB.

Recent strategies of drug delivery to brain without disrupting BBB can be classified into two types based on their energy (adenosine triphosphate (ATP)) requirements during the process: passive and active transport (Figure 4) [74]. Passive transport is an energy-independent process that lacks specificity. It includes the diffusion of small molecules through paracellular and transcellular pathways. Paracellular diffusion involves solute molecules moving between adjacent endothelial cells due to a negative concentration gradient. Only water-soluble molecules can pass through the paracellular space. In transcellular diffusion, non-ionic solute (molecular weight < 400 Da) with a desirable lipophilicity (e.g., hormones and steroids) can diffuse through the endothelial cells to brain [75]. However, in addition to the tight junction, some efflux pumps present at the luminal side of BCEC also limit the drug transport across BBB. Efflux pumps function in two phases, it initially inhibit cellular uptake of drug molecules in BCEC and later expel the drugs molecules (like doxorubicin, daunorubicin etc.) into blood against a negative concentration gradient in ATP dependent pathway [76]. P-glycoprotein (P-gp) is an example of efflux pump that plays a role in drug resistance in tumors. Regulating efflux pumps at the BBB represents another strategy for drug delivery to brain tumors. It is important to note that efflux pumps, although beneficial for protecting the healthy brain from harmful neurotoxins, can also pose challenges in drug delivery to brain tumors.

The active transport pathway often exploits endogenous receptor or transporter proteins that are expressed on the luminal side of BBB. Active transport routes include receptor mediated transcytosis (RMT), carrier mediated transcytosis, adsorption-mediated transcytosis, and cell-mediated transcytosis, all of which require ATP. In RMT, particles cross BBB via interaction with specific receptors expressed on apical surface of BCEC. It is an important pathway and is widely being explored for delivery of macromolecular biopharmaceuticals (e.g., protein or recombinant peptide-based therapeutics) or nanocarrier-mediated drug transport to the brain. The mechanism of RMT centers on endocytosis, where a ligand selectively binds to a receptor. This binding leads to the creation of an intracellular vesicle through membrane invagination. These vesicles are then transported and fused with the basolateral membrane, subsequently releasing the payloads as they detach from the membrane. It is worth mentioning that, like general endocytosis, in addition to the transcytosis from blood to brain some vesicles undergo lysosomal degradation while some others are recycled to the apical side in RMT. This process often targets specific receptors, including transferrin receptors, low-density lipoprotein (LDL) receptors, and insulin receptors for drug delivery to the brain.

Carrier- or transporter- mediated transcytosis (CMT) represents another dynamic active transport mechanism across the BBB, facilitating the transportation of essential nutrients such as amino acids and glucose into the brain. Nutrient molecules bind to the specific transporter proteins on the luminal side, causing conformational changes that enable the transfer of these nutrients into the brain. Glucose transporter isoform (GLUT-1) and large amino-acid transporter (LAT) are examples of such transporter. Small molecule drugs like L-DOPA and gabapentin utilize CMT to reach the CNS. However, the high specificity required for the interaction between transporters and ligands in this process limits its applicability in transporting macromolecular therapeutics [77]. Charged particles such as nanocarriers, predominantly traverse the BBB through adsorptive mediated transcytosis (AMT), relying on the electrostatic interactions between the negatively charged cell surface of BCEC and the particles [78]. Such interactions are non-specific, and many nanocarriers can be delivered; however, this is not devoid of the non-specific accumulation in other organs under systemic circulation. Cell-mediated transcytosis, which utilizes blood cells capable of BBB crossing for delivering drug to brain, has recently emerged as biomimetic strategy. In this method, immune cells or platelets are incorporated with drug-loaded nanocarriers which then cross BBB and navigate towards the inflammation sites within the brain by responding to chemotaxis signals and undergoing diapedesis [79]. More recently, extracellular vesicles, e.g., exosomes, have attracted significant attention as biomimetic drug carrier for CNS drug delivery. In addition, viral vectors have shown promise for gene delivery to brain. Further nanocarrier-mediated approaches have gained significant interest for efficient delivery to brain.

#### 3.2.1. Nanocarriers Mediated Drug Transport across BBB

Nanoparticles (NPs) such as liposomes, polymeric NPs, inorganic NPs, etc., are being used as drug carriers for decades [80,81,82,83,84,85]. Drug loading in NPs enhances circulation life of hydrophobic drugs in blood, protect nucleic-acid-based therapeutics from serum nucleases, or reduce the adverse off-target effects of drugs [86,87,88,89,90]. Surface of NPs can be engineered with PEG to achieve longer circulation life or with cell-penetrating peptide to enhance cellular uptake [91], or with targeting ligand to selectively deliver the payloads at targeted tissue [92]. Furthermore, drug release at the targeted tissue can be externally controlled by using stimuli-responsive nanocarriers [82]. Over the past few decades, many nanocarriers with size range ~10–300 nm have been explored for delivering small molecules, nucleotides, peptides, or proteins-based therapeutics to brain for combating various CNS diseases including brain tumor, neurodegenerative disorders, neuroHIV, stroke, etc. [82,88,89,90,93,94,95,96,97,98,99,100,101,102,103]. Such nanocarriers can cross the BBB by passive diffusion or can be engineered with some ligand at their exo-surfaces actively targeting some endogenous receptor/transporter protein on the BBB. For instance, liposomal encapsulation of cytotoxic anti-neoplastic agent doxorubicin has significantly mitigated the adverse effect of systemic chemotherapy as indicated by the enhanced safety index in a phase I trial involving 13 children with recurrent/refractory high-grade glioma (NCT02861222) [104]. Similarly, liposomal encapsulation of irinotecan has improved the safety profile of systemic chemotherapy in another phase I study with 34 high-grade glioma patients (NCT02022644) permitting its progression for Phase II trial [105]. However, although such encapsulation of cytotoxic chemotherapeutics improved the safety index of systemic chemotherapy in patients, the efficacy of nanoformulations might be facilitated by their passive accumulation via compromised integrity of the BBB around high-grade tumors. Clearly, there is a need for an active transport mechanism across the BBB for delivering drugs to combat low-grade tumor or other CNS diseases with intact or less compromised integrity of BBB.

Active targeting of receptor or transporter proteins expressed in brain capillary endothelial cells (BCEC) is the most widely explored nanocarrier-based drug delivery strategy across BBB. In this method, nanocarriers are surface engineered with targeting ligands of such receptors/transporters to deliver payload in brain via RMT or CMT which has been reviewed elsewhere in detail [106,107]. Although Transferrin, LDL family receptors (LDLR), insulin, and integrin receptors are widely explored receptors due to their high receptor-ligand affinity, GLUT and LAT-1 are some transporter proteins that are explored for drug delivery to brain.

Transferrin Receptor: Transferrin receptors (TfRs) control iron homeostasis via their natural ligand transferrin. TfRs are highly expressed in the luminal side of BBB and in brain tumors which makes them an attractive target for drug delivery to the brain [108]. Different TfRs ligands such as transferrin (Tf) itself (~80 kDa) [109], antibodies or antibody fragments [110], and peptides [111,112] are explored to examine their brain targeting efficacy by grafting such ligands with the biopharmaceuticals or at the exo-surface of nanocarriers which is reviewed in detail elsewhere [113]. For instance, Lam et al. have developed a transferrin-functionalized PEGylated liposomes for simultaneous delivery of temozolomide (TMZ) and bromodomain inhibitor in brain tumor. The combined chemotherapy regimen overcome the drug resistance of TMZ, reduced the tumor size, and improved the survival of mice with glioma compared to control groups, all while showing minimal systemic drug toxicity [109]. To overcome the plausible inhibition of RMT by competitive binding of endogenous Tf, nanocarriers are also surface engineered with monoclonal antibody (mAb), or peptide fragments targeting TfR. For instance, Yue et al. has conjugated OX26 antibody, a monoclonal antibody against TfR1, with micelles to develop an immunomicelle which shows high BBB-crossing ability [110]. A TfR specific heptapeptide T7 (HAIYPRH) with high binding affinity (K_d_ = 10 nM) has also been explored to target nucleotides and neoplastic drugs in glioma tissue in preclinical model [111,112]. Although such studies are at the preclinical stages, some have shown initial clinical promise [95]. For instance, a fusion of lysosomal enzyme iduronate 2- sulfatase (IDS) with anti- TfR antibody (JR-141) enabled successful delivery of the fusion protein into the CNS of patients with Hunter Syndrome under systemic settings (i.v.) in a phase I/II trial (NCT03128593) which shows promising therapeutic efficacy with no significant safety issue [114]. Notably, the use of TfR-targeting Tf-toxin conjugates has demonstrated clinical potential in anti-glioma therapy. Human Tf is linked to a diphtheria toxin featuring a CRM107 point mutation, resulting in the creation of Tf-CRM107. This conjugate displayed tumor growth inhibition when administered directly into the tumor in a U251 mouse model [115]. Subsequently, a phase I study following intra-tumoral injection revealed no adverse effects, leading to a phase II study involving patients with recurrent high-grade brain tumors. Although 35% of the patients displayed positive tumor responses and improved survival, the phase III was discontinued due to probable CNS toxicity with an indication for more targeted delivery of the toxin [116].

The sub-optimal clinical efficiency of TfRs targeting may be related to the high recycling rate (~90%) of endocytosed TfRs by BCEC to the luminal side as indicated by studies in mouse brain [117] where only 10% of TfR-NPs are able to reach brain parenchyma. Efforts to improve rate of transcytosis via varying ligand density on nanocarrier [118] or increasing receptor-ligand affinity are being examined [119]. Bivalent TfR antibodies with high receptor-affinity diverts the trafficking into lysosomes and subsequent degradation of the therapeutics indicating requirement of optimum receptor-ligand affinity for effective transcytosis [120,121]. Furthermore, interspecies variation of receptors, such as 2.5 times higher expression level of TfRs in mouse brain microvessels compared with that in human also contributes to the reduced efficacy during clinical translation of such active targeting strategies. Finally, ubiquitous expression of TfRs in other organs (liver, spleen, and bone-marrow) and uptakes of drugs in non-peripheral tissues also contribute to the compromised therapeutic efficacy of such targeting strategy [118].

LDL family receptors: LDL receptor (LDL-R) and receptors for LDL-R related proteins (LRP) are the most explored among LDL family receptors for drug delivery to CNS. This is a class of receptors that help lipid transport to the brain [122] and are expressed in CNS cells, BBB endothelium, and upregulated in cell surface of glioma cells [123,124]. Apolipoprotein B (ApoB) and ApoE are the ligands of LDL-R. ApoE, which is prevalent in the brain, especially in CSF and plasma, has been widely explored for drug delivery to brain. LDL-R mediated transcytosis of NPs are generally two types which rely on (i) avidity-based surface attachment of ApoE to NPs or (ii) surface-functionalization of NPs via conjugation with ApoE or its derivatives. For instance, poly-butylcyanoacrylate (PBCA) NPs coated with surfactant polysorbate 80 (PS-80) [125] show enhanced cellular internalization of the NPs by 20-fold in human and bovine endothelial cells compared to the uncoated NPs [126]. Later mechanistic study reveals that this surface coating with PS-80 enables adsorption of plasma ApoE onto NPs which is then recognized LDL-R expressed in BCEC and undergoes RMT to brain parenchymas [127]. Study indicates that such receptor–ligand interaction is strong to exclude the size-effect of NPs in the BBB crossing when examined with NPs of varying sizes from 87 nm to 464 nm [128]. Such PS-80 coating approach to enhance brain delivery has also been explored for other nanoparticles (e.g., polylactic acid (PLA), solid lipid nanoparticles (SLN), in different combination of therapeutics which has been reviewed in detail elsewhere [129]. In the second strategy, synthetic peptides containing short binding sequence to LDL-R has shown some pre-clinical promise. For instance, Grafals-Ruiz et al. designed gold-liposome nanocarriers with ApoE peptides on their exo-surface for the systemic delivery of small-nucleic acids to the brain of mice with gliomas [130].

LDL-R related proteins (LRP), especially LRP1 has also been targeted for RMT-based drug delivery to brain due to its high expression level on human BBB which is comparable to TfR and insulin receptors [131,132]. Particularly, Angiopep-2 peptide has gained enormous attention as targeting ligand for LRP1 mediated drug delivery to brain tumor in pre-clinical study [133,134,135]. In a phase I clinical investigation involving ANG1005 (Angiopep-2 peptide conjugated to paclitaxel), the progression of disease was reduced among 8 out of 27 patients, leading to its advancement into a phase II clinical trial (NCT01967810) for patients with high-grade glioma [136]. Furthermore, ANG1005 has recently demonstrated clinical benefits in a phase II clinical trial aimed at treating patients with recurrent brain metastasis originating from breast cancer [137].

Insulin receptors (IR) and insulin-like growth factors receptor 1 (IGFR-1), which are expressed in the brain and BBB, are also explored for drug delivery to brain. For instance, intravenous infusion of enzyme laronidase fused to an IR binding antibody (AGT-181) in a phase II trial (NCT03053089) to treat Hurler syndrome (alternatively known as mucopolysaccharidosis type I) have shown well-tolerated safety profile with satisfactory efficacy in pediatric patient [138].

Cell adhesion molecules (CAMs) such as integrin, selectin, and gap junction proteins connexin have also been explored as drug delivery target to brain [139,140,141,142,143]. For instance, the use of paclitaxel (PTX) loaded nanoparticles targeting integrin, specifically PTX-c(RGDyK)-NP based on poly(trimethylene carbonate), extended the survival of U87MG glioma-bearing Balb/c mice by 22 days compared to free PTX [144]. Similarly, Nukolova et al. used nanogels conjugated with a monoclonal antibody of connexin 43 (Cx43), a gap junction protein, to deliver cisplatin in C6 gliomas. This approach was reported to significantly enhance the survival of animals [145].

Other Receptor and Transporter-Mediated Targeting Systems: Other than the afore-mentioned receptors, many other endogenous receptors, or transporters on the BBB such as acetylcholine receptor (nAChRs), glutathione (GSH) transporter, GLUT, LAT-1, etc., are being explored for the delivery of drugs to the brain using nanocarriers. For instance, Chaudhuri and co-workers have developed a nAChRs receptor-targeted liposomes by decorating with nicotine at their exo-surface [146]. They also designed another liposome that target LAT-1, with L-DOPA grafted onto the surface [147], to transport the STAT-3 inhibitor WP-1066 in mouse brain tumor. Overall, such active targeting strategies hold significant promise for macromolecular pharmaceuticals like recombinant proteins, although efficacy of such nanocarriers mediated RMT is still in pre-clinical stages.

#### 3.2.2. Magnetic Field Assisted Crossing of BBB

Application of an external magnetic field is another physical method for drug delivery to the brain which not only spatially guides the magnetic nanoparticle to the targeted region but also significantly improves the speed and time for drug delivery. In this method, paramagnetic nanoparticles (PMNP), especially superparamagnetic iron oxide nanoparticles (SPIONs) with sizes ~10–100 nm, are used. Although magnetic nanoparticles (MNPs) and liposomes in diameters of 70 nm do not cross the BBB, the application of a static magnetic field facilitates its delivery across BBB. Particle size controls the magnetic susceptibility under a fixed static magnetic field. Small SPIONs exhibit higher magnetic susceptibility (highest with crystalline domain with 10–30 nm) than larger paramagnetic nanoparticles containing many crystalline domains mutually diminish the net magnetic moment. In addition, nanoparticles of 10–100 nm are considered optimum due to their longer systemic circulation times. The size of the nanoparticles determines their effect on BBB. For instance, SPION with ~117 nm under 0.39 Tesla did not disrupt BBB integrity whereas magnetic nanoparticles with a size of 800 nm cause leakage in BBB under the same magnetic field strength. In addition, the lower particle size with higher magnetic susceptibility requires less field strength, although no adverse effect in cells is reported with the static magnetic field as strong as up to 10 Tesla. Although the transcellular migration through BCEC via uptake or nanoporation is presumed to be the major pathway, some recent studies indicate interaction of SPIONs with junction proteins such as VE-cadherin may contribute to additional involvement of the paracellular pathway for BBB crossing [148,149].

**Figure 5:**
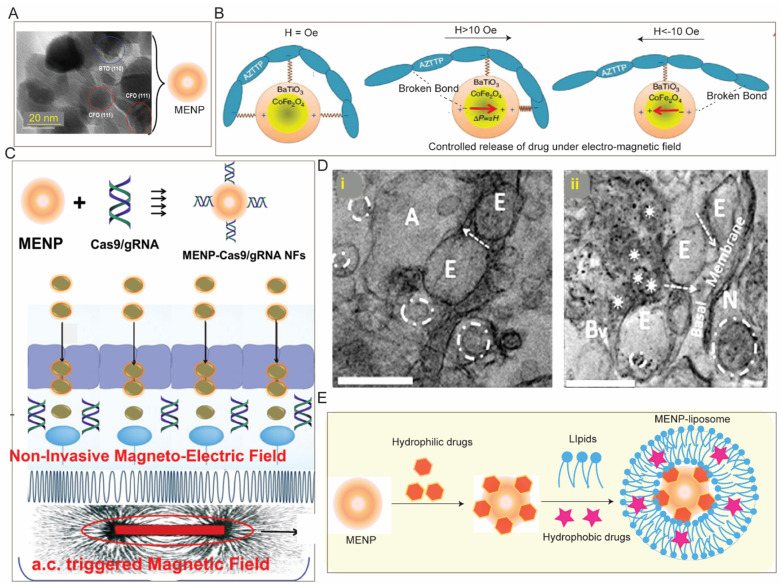
Magnetic field assisted drug delivery to the brain: (**A**) TEM of a magneto electric nanoparticle (MENP) containing CoFe_2_O_4_ at the core with a shell of BaTiO_3_ (**B**) which enables controlled drug release under alternating electric stimuli, (**C**) MENP (~30 nm) can be loaded with CRISPR via hydrophilic interaction which allows their non-invasive delivery across BBB (in vitro) under electromagnetic field, (**D**) TEM of brain tissue from (**i**) untreated mice and (**ii**) mice intravenously administered with 10 mg/kg BW of MENP indicating brain accumulation of MENP (black dot in **D**(**ii**)). (**E**) Schematic for potential simultaneous delivery of hydrophilic and hydrophobic payload to the brain using MENP-liposome composed of a lipid coating embedded with hydrophobic drug onto the hydrophilic drug-loaded MENP. Adopted with permission from Refs. [148,157,158,161].

SPIONs are used in clinics for MRI as a contrast agent and hold potential for other biomedical purposes including targeted drug delivery, image-guided drug delivery, hyperthermia, etc., for the management of CNS diseases [96,150,151]. SPIONs can be surface-functionalized with different polymers, lipids etc. to achieve desired drug loading or pharmacokinetic property. For instance, polystyrene-coated SPIONs (~100 nm) under 0.1 T external magnetic field cross the BBB, accumulate in the brain parenchyma, and exhibit 25 times greater retention with minimal neurotoxicity. Similarly, transferrin-coated PEGylated magneto liposomes (~130 nm) exhibit complete transmigration across an in vitro BBB under 0.08 T magnetic field without affecting the BBB [152]. Beyond small molecule anti-cancer drugs [150], magneto liposomes also have been used to facilitate delivery of therapeutic peptide [153], brain-derived neurotrophic factor (BDNF) [154] or antiretroviral agents across the BBB [155,156]. For instance, to enhance the BBB permeation of antiretroviral agent 3′Azido-3′deoxythymidine-5′-triphosphate (AZT), it is complexed with SPIONs (~25 nm) followed by coating with liposome. This magneto liposome containing encapsulated AZT (~150 nm) crosses the BBB (in vitro) under 0.3 T field and results in three times higher accumulation of AZT across BBB compared to the only AZT [155]. Importantly, to further gain control for on-demand drug release, MNP are modified to electromagnetic nanoparticles (MENP) [157] which exhibit brain accumulation under low ac magnetic field with no adverse effect in rodents [158] and non-human primates [159] and can facilitate delivery of hydrophilic therapeutics including siRNA [160], CRISPR [161] across in vitro BBB (Figure 5). It is worth mentioning that such MENP can also be used for non-invasive deep brain stimulation to control neuroactivity in Parkinson’s disease [162]. Many studies have claimed lysosomal degradation of SPIONs as histopathological evaluation of major organs involved in systemic circulation revealed no iron-positive pigment or related macrophage accumulation [158,163]. However, some recent studies have reported toxicity of SPIONs as it causes an imbalance in iron homeostasis which may induce oxidative stress and inflammation leading to genotoxicity due to its differential interaction with mitochondria [164,165]. Clearly, in-depth evaluation of in vivo toxicity in long-term exposure to SPION is needed.

#### 3.2.3. Cell-Based Biomimetic Strategy of BBB Crossing

Bioinspired carriers such as blood cells, cell-membrane-coated nanocarriers, exosomes, etc., are being explored recently for drug delivery across BBB due to their longer circulation life and biocompatibility [79]. Leukocytes such as macrophages, monocytes, and neutrophils are most explored for brain delivery due to their inherent chemotactic recruitment property, especially brain diseases with inflammation. In such methods, drugs are first loaded in nanocarriers which are then incorporated into cells to facilitate delivery across the BBB. For instance, Xue et al. have used neutrophils to deliver paclitaxel loaded liposomes in residual tissue post-surgery which have suppressed the recurrence of glioma growth [166]. To treat ischemic stroke, Xu et al. have developed a ‘nanoplatelet’ by coating a neuroprotective agent loaded dextran-based nanocarrier with platelet membrane surface-engineered with thrombin-responsive anti-ischemic drug and TAT peptide. This ‘nanoplatelet’ crosses the BBB, clears the thrombus clog at the ischemic site in the brain and delivers neuroprotective agent to combat ischemic stroke [167]. In another study, to combat encephalitis Yuan et al. have utilized a macrophage-derived exosome for delivering brain-derived neurotrophic factor (BDNF) to inflamed brain [168]. Such BDNF-loaded exosomes crosses BBB via intercellular adhesion molecule 1 (ICAM-1) which is upregulated under encephalitis-related inflammation. However, cell-based carriers suffer from some common limitations such as viability of cell-based carriers arising due to leaching of drug from nanocarriers. In addition, there are some cell-specific limitations like risk of immune activation while using leukocytes or activation of platelets while using it as drug carrier that may cause undesired thrombosis or bleeding. Exosomes are extracellular vesicles which show ~0.5% passive brain accumulation and have attracted attention for drug delivery to brain and treating neurological conditions. For instance, i.v. administration of dopamine-loaded exosomes enhanced the dopamine levels (15 times) in mouse brain [169]. Further understanding of the interaction between the BBB and bio-mimetic carriers are necessary for proper engineering of such carrier to maximize therapeutic benefit. Extracellular vesicles (EVs) derived from the cells have been explored for neuroprotective applications including traumatic brain injury. In one example, the EVs derived from mesenchymal stromal cells (MSCs) were examined from neuronal cell protection using in vitro models [170].

#### 3.2.4. Viral Vector for Drug Delivery to Brain

Neurotropic viruses that can specifically infect the brain are currently being explored as drug carriers to the brain. For instance, adeno-associated viruses (AAVs) and lentivirus which have been widely used in gene therapy, are being explored for BBB trafficking. AAVs can stably transduce genes to CNS cells, e.g., neurons, astrocytes, BCECs, oligodendrocytes, and ependymal cells, and are observed to retain their expression in pre-clinical models (longer than 6 years in monkeys) [171,172]. Despite the shortcomings of strict packaging limit (~4.7 kb) [173] and pre-existing immunity in certain serotypes [174], the transduction efficacy and tolerability [175,176] of some serotypes, e.g., AAV9, AAV2 have affirmed their emerging exploration in different pre-clinical CNS disease models [177]. Importantly, the recent FDA approval of Zolgensma which is the first AAV-based (AAV9) gene therapy for spinal muscular atrophy type 1, has created a milestone for viral vectors mediated gene therapy for CNS diseases with many other ongoing clinical trials.

The packaging capacity can be improved by using lentiviral vectors which possess a bigger packaging capacity. However, the tendency to integrate with host gnome and thereby, potential adverse effect by insertional mutagenesis in CNS target cells has greatly limited its clinical scope [178,179]. For instance, even the non-integrating lentivirus also have residual propensity to integrate with the host genome which may have adverse effect due to insertional mutagenesis in CNS target cells. This effect can be minimized by using ex vivo transduction of cells; however, the efficacy of such lentiviral-based therapy will further depend on CNS entry efficiency of the transduced cells.

Despite the recent clinical success, application of viral vectors is greatly limited to gene therapy and often requires invasive mode of administration. Efforts to improve BBB permeability using different serotypes including AAV8, AAV9, and AAV10 have not shown satisfactory outcomes yet, as such neurotropic viral vectors poorly transduced BCECs [180,181]. In addition, a high dose of such vectors may be required for BBB crossing which may create challenges like autoimmunity risk and reduced rate of BBB transport due to neutralization of such serotypes by their pre-existing antibodies [182]. Finally, factors like vector purity, self-inactivating or non-integrating vectors, transgene sequence, etc., which influence their therapeutic efficacy, are under investigation, more studies are required for robust safety assessment in selecting an optimal viral vector.

## 4. Localized Drug Delivery Strategies

To bypass the hurdle of BBB crossing and enhance therapeutic efficacy of drugs, localized drug delivery strategies such as injections, convection enhanced delivery (CED), and administration of implants are developed. Although these strategies are invasive, site-specific delivery of the therapeutics with high bioavailability and minimal drug loss can be achieved.

### 4.1. Injection

In this method drugs are directly injected or infused into the disease site or remaining cavity after the resection of the tumor which is less toxic and much effective than systemic administration. However, this method is not a breakthrough for treating CNS disease due to their high risk of side effects such as edema, infections, and backflow of drugs into the catheter, narrow drug distribution in the injection site [183].

### 4.2. Convection Enhanced Delivery (CED)

To prevent the backflow of drugs and improve the drug distribution in the brain tissue, CED is developed [184]. In this method, an implantable pump is connected to the catheter to maintain a convective flow. Usually, the catheter is introduced stereotactically while the constant pressure from the pump maintains a convection flow (independent of drug diffusivity) of the drug solution into the delivery site. In addition, the convective flow allows the drug solution to cover longer distance in the brain compared to direct injection/infusion [185]. CED can be further connected to a real time magnetic resonance imaging method to monitor the drug distribution [186]. CED has been widely tested for the delivery of a broad spectrum of therapeutic agents, including small molecules [185,187,188], macromolecules [189,190], nanocarriers [191,192], and immunotoxins [193]. CED for delivering drug from liposomal and polymeric nanocarrier is of importance which can offer many advantages like less systemic side effects, sustained drug release, and larger distribution with targeted delivery. CED is a real progress in the field of infusion mediated drug delivery to the brain; however, despite its significant promise, invasiveness, and the common side effects of infusion methods, such as infection and edema, are common limitations. The safety and efficacy of this method is yet to clearly determined as it fails to meet clinical end points in many Phase III trials [194].

### 4.3. Implants

**Figure 6:**
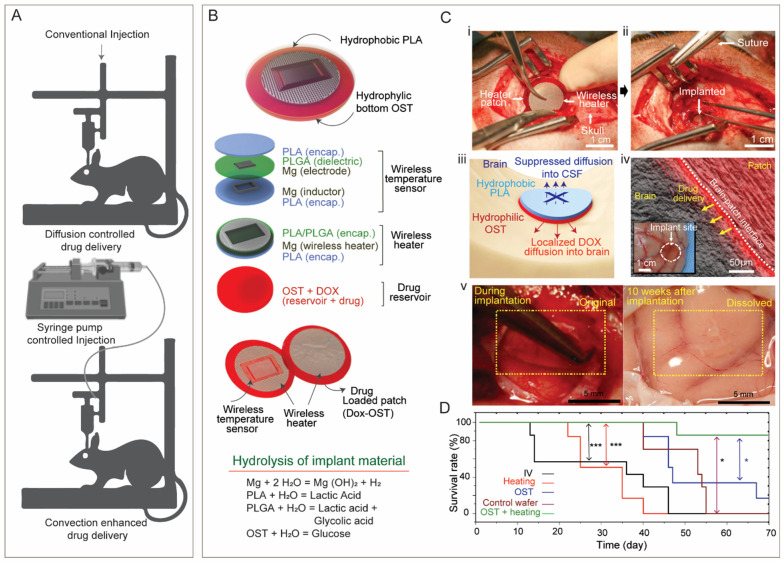
Localized drug delivery in the brain: (**A**) Schematic for conventional injection and convection-enhanced stereotactic drug delivery method; (**B**) a biodegradable wireless electronic patch (1 cm) made of tissue adhesive bifacial soft polymeric material PLA and OST and containing a temperature sensor, heater, and drug reservoir for intra-cranial local drug delivery via thermic actuation, Stereotactic implantation of the patch into brain (**C**, **i**,**ii**) allows local drug delivery and prevention of drug diffusion to CSF (**C**, **iii**), tissue assimilation (**C**, **iv**) and resorption with host tissue (**C**, **v**). Sustained drug delivery from this wireless patch under thermic actuation (OST + heating group) enhances the survivability of glioma-bearing mice compared to control groups including OST (device without actual), control wafer (custom Giladel), and heating (actuation with the empty patch). (**D**) Kaplan–Meier survival rate plots of the indicated treatment group in the mouse model, * *p* < 0.05, *** *p* < 0.001 by log-rank test with Bonferroni correction. Adopted with permission from refs. [183,198] under the CC-BY license, version 4.0.

Polymeric implants including wafers, gels, microspheres, and nanospheres have been tested for localized delivery of therapeutics in brain in different shape and sizes [183]. Among these, wafers attracted attention since FDA-approval of Gliadel in 1996 [195,196]. Wafers are drug loaded polymeric implants that look like coins in shape and size. Wafers are implanted in the remaining cavity post-surgical resection of a tumor where they act as platforms for sustained drug release. The safety and effectiveness of Giladel, a wafer made of co-polymer of phosphino carboxylic acid and sebacic acid and loaded with chemotherapeutics BCNU (1,3-Bis(2-chloroethyl)-1-nitrosourea) alone and in combination with radiotherapy led to its FDA-approval in treating recurrent and newly diagnosed glioma [196,197]. Since then, various other polymers like poly(lactide-co-glycolide), poly(vinyl acetate) extrudates, etc., in combination with different chemotherapeutics are tested in various trials, though only Gliadel^®^ is legally approved so far for the treatment of malignant gliomas. However, the major drawbacks of wafers as standard delivery platform in treating glioma are lack of deep tissue penetration of therapeutics which is crucial for invasive tumor like glioma. The low diffusivity of the therapeutics allows drug distribution only over a few millimeters from the delivery site. In addition, leakage of the drug into the CSF, adverse neurological complication arising from mechanical mismatch of the patch, control over the spatial and temporal drug release, biodegradability, and tissue adhesiveness of the patch, etc., need attention to improve therapeutic efficacy. Efforts are being made to overcome such limitations. For instance, Lee et al. [198] have developed an adhesive, flexible, bioresorbable device covering a drug-loaded patch integrated with wireless electronics for intracranial drug release via mild-thermic actuation (Figure 6B,C). The thermic actuation in presence of an alternative magnetic field allows deep tissue penetration while preventing leakage towards CSF (Figure 6C(iii)). The softness and optimum hydrophobicity/hydrophilicity in the bioresorbable device material containing PLA layer (on top) and oxidized starch (OST) at bottom layer (Figure 6B) allow conformal adhesion and assimilation to the host target brain tissue over time (Figure 6C(iv,v)) while minimizing the neurological complication of rigid implant. Other than wafers or large implants, gels and micro/nanosphere-based or polymeric microchips have been tested as alternate drug delivery implants; however, none of these are clinically approved and share common limitation of lack of deep tissue penetration of therapeutics [183].

### 4.4. Intranasal Delivery (IN)

Delivery of therapeutics to the brain through nasal route is another non-invasive approach to circumvent the BBB. Although the mechanism of intranasal drug delivery to brain is not well understood, the olfactory/trigeminal pathway is presumed to be the significant route for nose-to-brain drug delivery [199]. The drug molecules into the nasal cavity first experience the mucociliary clearance in the vestibular region and afterwards move to the posterior regions of the nasal cavity respiratory region and the olfactory region from where drug molecules are transported to mid-brain and brainstem along the trigeminal and olfactory neurons, respectively. Then, drug molecules are distributed in other regions of the brain via convection flow or perivascular routes. Some clinical trials of IN for the management of neurological disorders including Alzheimer’s diseases, Parkinson’s disease, etc., are ongoing [200,201]. Mucosal clearance, limited dosing volume (100–150 μL or 20–50 mg powders), and metabolic stability of drugs against nasal cavity enzymes are the limiting factors. Mucoadhesive polymeric nanoparticles and non-irritant drug formulations may hold promises to overcome such limitations; however, further research may be needed.

## 5. Conclusions and Future Perspective

With the growing number of neurological disorders in the aged population and brain tumors, there is an urgent need for safe and efficacious targeted drug delivery to brain. Despite years of efforts in CNS drug development, recent FDA approval of Zolgensma, viral-based gene therapy, is the first approved BBB-crossing biologics. However, the invasive mode of administration of such therapy induces vulnerability towards infection by neurotoxins or other pathogens. In addition, viral vectors are limited to gene therapy which is only effective for diseases with single gene mutation. In an era of biopharmaceuticals such as recombinant proteins being the most approved drugs for other diseases, its scope is greatly limited by poor delivery of such macromolecules across BBB which reflect the poor rate of clinical translation (~8%) of CNS drugs. With the vast spectrum of CNS drug delivery strategies, each having distinct pros and cons, it may be challenging to establish a universal platform technology for CNS drug delivery. In addition, with recent advances in tissue engineering efforts are also being made to overcome the gap in interspecies tissue homogeneity. Nanotechnologies have demonstrated enhancements in BBB permeability, region-specific targeting, drug stability, and delivery. Nevertheless, RMT and CMT have shown initial promises in delivery of macromolecular biopharmaceuticals and small molecules, respectively, across the BBB. However post-discovery, challenges include navigating regulatory hurdles, managing costs, and ensuring accessibility of these new technologies. Overall, more efforts should be invested in CNS drug delivery technologies in parallel to drug development, and fostering concentrated efforts through industry–academia collaboration may lead to safe and effective drug delivery to the brain in the near future.
